# A Certain Liveliness in Blackburn

**Published:** 1918-07-20

**Authors:** Nathan A. Smith

**Affiliations:** General Superintendent and Secretary. Blackburn and East Lancashire Royal Infirmary


### THE EDITOR'S LETTER-BOX.

### A Certain Liveliness at Blackburn.

*To the Editor of* The Hospital.

Sir,—Some time ago you referred in the columns of The Hospital to the contributions from this district in support of this institution, and gave comparative figures as to the support given to similar institutions from workpeople in their respective districts.

Recently his Worship the Mayor of Blackburn, Colonel L. Cotton, J.P., convened a public meeting of representatives from all trades throughout Blackburn and East Lancashire with a view to the adoption of a system of weekly and fortnightly contributions from the workpeople in aid of the Royal Infirmary. I beg to enclose reports of the meetings, extracts from which will probably be interesting to hospital committees.

At the public meeting held in the Blackburn Town Hall on Tuesday evening last week, the following resolution was unanimously passed :—

"That this meeting is in favour of a scheme of voluntary systematic weekly or fortnightly contributions from all classes of labour in the Blackburn and East Lancashire district towards the Royal Infirmary, and such other charitable institutions as the committee governing the fund think fit, and that a special committee consisting of five members of the infirmary board of management, one from the Blackburn District Convalescent Home and one from the District Nursing Association, and one representative from *each* trades union in Blackburn and district, with power to co-opt other representatives, be, and is hereby appointed to consider this scheme and put it into operation as early as possible."

A sub-committee was appointed to formulate and carry out the scheme.—I am, yours faithfully,

Nathan A. Smith,
General Superintendent and Secretary.

Blackburn and East Lancashire Royal Infirmary,
July 13, 1918.

[We deal with the new scheme in our Notes this week.—Ed. The Hospital.]

